# Reduced fertility caused by meiotic defects and micronuclei formation during microsporogenesis in x*Brassicoraphanus*

**DOI:** 10.1007/s13258-021-01050-x

**Published:** 2021-02-08

**Authors:** Hosub Shin, Hye Rang Park, Jeong Eun Park, Seung Hwa Yu, Gibum Yi, Jung Hyo Kim, Wonjun Koh, Hyun Hee Kim, Soo-Seong Lee, Jin Hoe Huh

**Affiliations:** 1 Department of Agriculture, Forestry and Bioresources, Seoul National University, Seoul, 08826 South Korea; 2 Interdisciplinary Program in Agricultural Genomics, Seoul National University, Seoul, 08826 South Korea; 3 Plant Genomics and Breeding Institute, Seoul National University, Seoul, 08826 South Korea; 4 Department of Life Science, Chromosome Research Institute, Sahmyook University, Seoul, 01795 South Korea; 5 BioBreeding Institute, Anseong, 17544 South Korea; 6 Research Institute of Agriculture and Life Sciences, Seoul National University, Seoul, 08826 South Korea

**Keywords:** Intergeneric hybrids, Pollen fertility, Meiosis, Micronucleus, x*Brassicoraphanus*

## Abstract

**Background:**

Hybridization and polyploidization events are important driving forces in plant evolution. Allopolyploids formed between different species can be naturally or artificially created but often suffer from genetic instability and infertility in successive generations. x*Brassicoraphanus* is an intergeneric allopolyploid obtained from a cross between *Brassica rapa* and *Raphanus sativus*, providing a useful resource for genetic and genomic study in hybrid species.

**Objective:**

The current study aims to understand the cause of hybrid sterility and pollen abnormality in different lines of synthetic x*Brassicoraphanus* from the cytogenetic perspective.

**Methods:**

Alexander staining was used to assess the pollen viability. Cytogenetic analysis was employed to monitor meiotic chromosome behaviors in pollen mother cells (PMCs). Origins of parental chromosomes in x*Brassicoraphanus* meiocytes were determined by genome in situ hybridization analysis.

**Results:**

The x*Brassicoraphanus* lines BB#4 and BB#6 showed high rates of seed abortion and pollen deformation. Abnormal chromosome behaviors were observed in their PMCs, frequently forming univalents and inter-chromosomal bridges during meiosis. A positive correlation also exists between meiotic defects and the formation of micronuclei, which is conceivably responsible for unbalanced gamete production and pollen sterility.

**Conclusion:**

These results suggest that unequal segregation of meiotic chromosomes, due in part to non-homologous interactions, is responsible for micronuclei and unbalanced gamete formation, eventually leading to pollen degeneration and inferior fertility in unstable x*Brassicoraphanus* lines.

## Introduction

Hybridization and polyploidization are driving forces in plant evolution, providing phenotypic variability and competitiveness for adaptation (Chen 2007; Cheng et al. 2018; Soltis and Soltis 2009; Van de Peer et al. 2017; Wendel 2000). Polyploidy is predominantly observed in flowering plants (Masterson 1994), and many domesticated crop species, including oilseed rape (Chalhoub et al. 2014), mustard (Yang et al. 2016), wheat (International Wheat Genome Sequencing 2014), cotton (Zhang et al. 2015), and strawberry (Edger et al. 2019), have undergone polyploidization events. Although there are many spontaneous hybrids in nature, successful speciation through hybridization is not straightforward, owing to hybridization barriers that are frequently observed in early generations of hybrid offspring, manifested as outbreeding depression, reduced viability, and reduced fertility (Abbott et al. 2013; Todesco et al. 2016). Several mechanisms have been proposed to explain hybridization barriers such as abnormal endosperm development, self-incompatibility and meiotic failure during gametogenesis (Dion-Cote and Barbash 2017).

Meiosis is the process by which four haploid gametes are generated from a diploid progenitor cell for sexual reproduction. During this process, the progenitor cell is divided into the tetrad through two successive meiotic cell divisions, which entails strand exchanges between homologous chromosomes and precise segregation of sister chromatids, and for the production of male gametophytes, four microspores are released from the tetrad and develop into pollen grains. Therefore, successful meiosis during gamete formation is an essential step to produce fertile pollen in plant reproduction. In particular, abnormal interactions between non-homologous chromosomes may have detrimental consequences, including reduced pollen viability in allopolyploids. Many studies have reported non-homologous chromosome pairing in artificially synthesized allopolyploid plants such as rapeseed, tobacco and wheat (Chen et al. 2018; Xiong et al. 2011; Zhang et al. 2013). Unpaired univalents, homoeologous bivalents, or multivalents induced by aberrant chromosome pairings are likely to cause meiotic chromosomes to mis-segregate, resulting in impaired fertility (Cifuentes et al. 2010; Szadkowski et al. 2010).

**Fig. 1:**
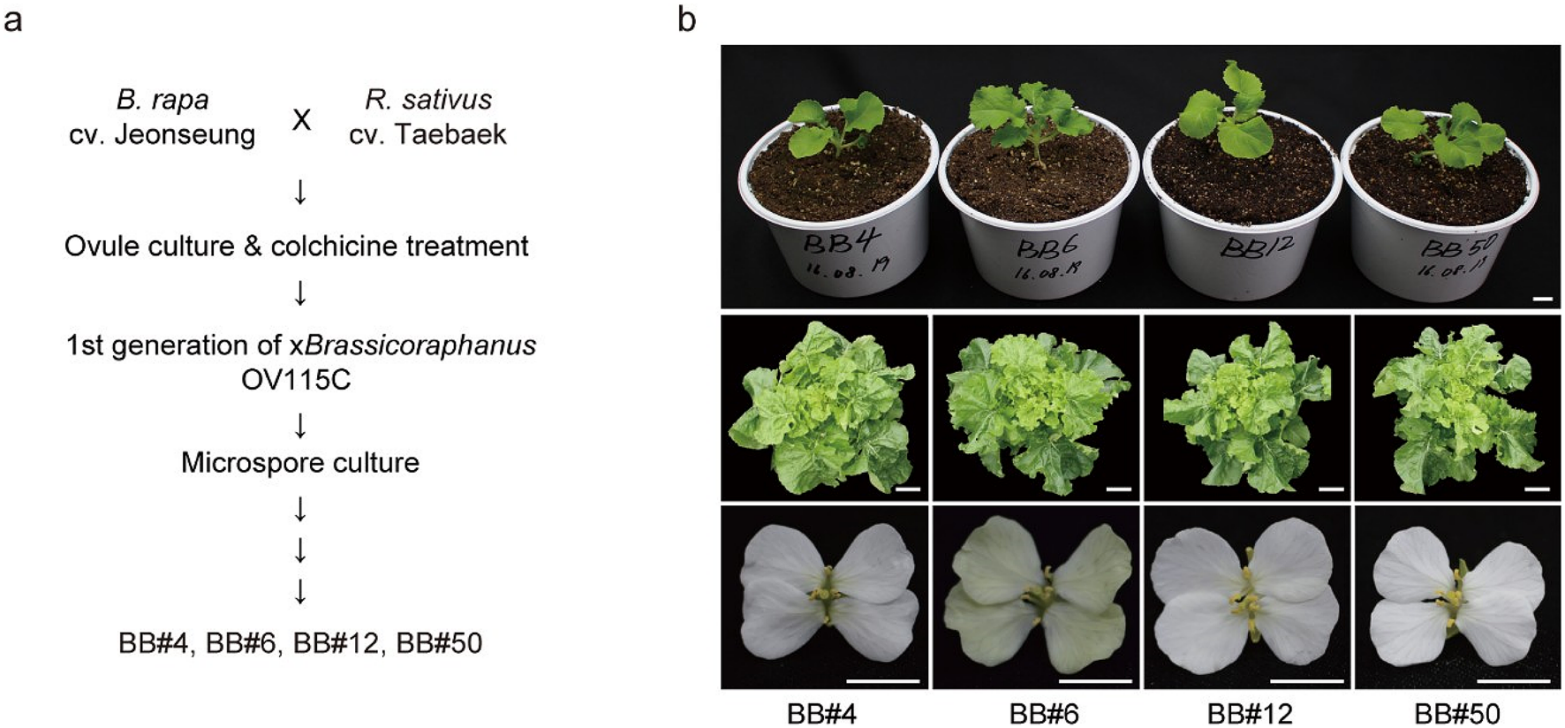
Pedigree and morphology of four x*Brassicoraphanus* lines. **a** The first generation of x*Brassicoraphanus*, OV115C, was obtained by a cross between *B. rapa* cv. Jeonseung and *R. sativus* cv. Taebaek. Four x*Brassicoraphanus* lines were developed from OV115C through microspore culture. **b** Morphology of seedlings (upper), mature plants (middle) and flowers (lower). Scale bar = 1 cm (flower and seedling) or 10 cm (mature plant)

The Brassicaceae family is well known for species diversification through allopolyploidization events between related species as described by the “U’s triangle” (U 1935), wherein three *Brassica* species *B. napus* (AACC; 2n = 4x = 38), *B. juncea* (AABB; 2n = 4x = 36) and *B. carinata* (BBCC; 2n = 4x = 34) emerged naturally by hybridization and polyploidization among three diploid species *B. rapa* (AA; 2n = 2x = 20), *B. nigra* (BB; 2n = 2x = 16) and *B. oleracea* (CC; 2n = 2x = 18). Besides interspecific hybridization, an intergeneric hybridization event may arise sporadically between two divergent species that belong to different genera. An exemplary case of such hybridizations is x*Brassicoraphanus*, an intergeneric allotetraploid synthesized from a cross between *B. rapa* and *Raphanus sativus*. The x*Brassicoraphanus* cultivar BB#1 was reported to display exceptional phenotypic uniformity and high fertility (Lee et al. 2002, 2011, 2017), and unlike many other synthetic hybrids, showed normal diploid-like meiotic behaviors (Park et al. 2020). There are several lines of x*Brassicoraphanus* besides BB#1. The four x*Brassicoraphanus* cultivars BB#4, BB#6, BB#12 and BB#50 were generated from a cross between *B. rapa* cv. Jeonseung (2n = 2x = 20) and *R. sativus* cv. Taebaek (2n = 2x = 18) (Lee et al. 2002). The first generation of x*Brassicoraphanus*, OV115C, was obtained by ovule culture and colchicine treatment (Fig. 1a) (Lee et al. 2002). From OV115C, lines BB#4 and BB#6 were generated by microspore culture, and BB#12 and BB#50 were developed by microspore culture with the treatment of *N*-methyl-*N*-nitroso-urethane (NMU).

Notably, lines BB#4 and BB#6 have severe pollen abnormality and seed abortion, which is reminiscent of a post-zygotic hybridization barrier often observed in many hybrid offspring. In this study, we compared seed yield, pollen shape and viability, tetrad formation, and meiotic chromosome behaviors in pollen mother cells (PMCs) among different x*Brassicoraphanus* lines. We frequently detected micronucleus formation at the tetrad stage in x*Brassicoraphanus* lines BB#4 and BB#6, from which more abnormal pollen was generated. In addition, univalent chromosomes and chromosome bridges were observed during microsporogenesis, presumably leading to micronucleus formation in the tetrad and pollen abnormality. Our data strongly suggest that unequal segregation of meiotic chromosomes during microsporogenesis is likely responsible for the formation of micronuclei and unbalanced gametes in a pollen tetrad, eventually leading to pollen deformation and reduced fertility in unstable x*Brassicoraphanus* lines.

## Materials and methods

### Plant materials and growth conditions

Seeds of x*Brassicoraphanus* cultivar BB#4, BB#6, BB#12 and BB#50 were sterilized in 50% hypochlorite solution with 0.1% Triton X-100 for 90 s, followed by 10 washes with sterile distilled water. The seeds were plated on Murashige and Skoog medium (Duchefa, Netherlands) supplemented with 2% (w/v) sucrose and 0.8% (w/v) plant agar (Murashige and Skoog 1962). Plates were incubated at 24 °C, with a daily cycle of 16 h of light and 8 h of dark, for 2 weeks. Plants were vernalized at 4 °C, with the same light cycle, for 4 weeks. Plants were transferred to soil in pots and placed in the glasshouse with the same light conditions.

### Alexander staining

Alexander's stain solution was prepared and used to distinguish between viable and nonviable pollen (Alexander 1969). Mature floral buds were collected and fixed in Carnoy’s solution (alcohol:acetic acid, 3:1, v/v), then placed on microscope slides and dissected to release the pollen. Stain solution (40–60 μl) was applied before the sample dried completely. After staining the pollen grains, a coverslip was placed over the sample and uniform pressure was applied. The slides were examined using an Axioskop2 microscope equipped with an Axiocam 506 color CCD camera (Zeiss, Germany).

### Scanning electron microscopy (SEM)

For SEM evaluation, properly dried pollen of each cultivar was dusted onto aluminum stubs with a thermosensitive glue. The sample on each stub was sputter-coated with a 30 mÅ- thick layer of platinum for 200 s at 20 mA, using a Sputter Coater (Leica, Austria). Pollen grains were observed using a field emission scanning electron microscope (Zeiss, Germany).

### Genome in situ hybridization (GISH)

Floral buds were fixed in the aceto-ethanol (1:3 v/v) solution for 24 h and stored at − 20 °C in 70% ethanol until use. The fixed buds were rinsed in distilled water. Slide preparation, probe preparation and hybridization were performed according to published protocols (Park et al. 2020). Slides were imaged using an Axioskop2 microscope equipped with an Axiocam 506 color CCD camera (Zeiss, Germany).

## Results

### Variability of seed formation among x*Brassicoraphanus* lines

**Fig. 2:**
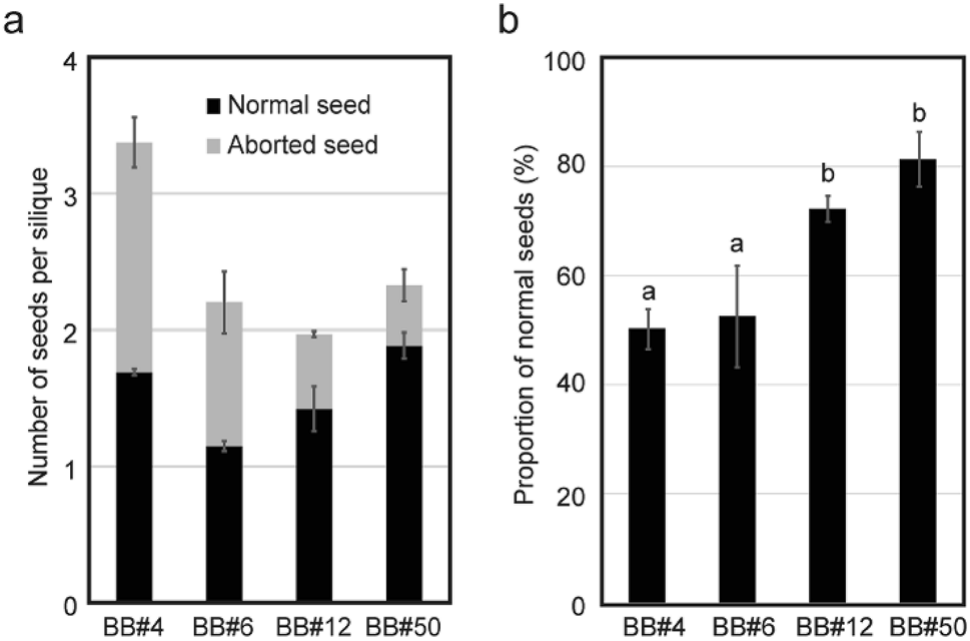
Seed counts per silique and proportions of normal seeds in four x*Brassicoraphanus* lines. **a** Seed numbers per silique. Black and grey bars represent the numbers of normal and aborted seeds, respectively. **b** Proportion of normal seeds per silique. More than one hundred siliques from two independent plants were analyzed for each line. Error bars indicate standard deviations and differences among x*Brassicoraphanus* lines were tested with one-way ANOVA, as indicated with letters, using Duncan’s multiple test (*p* < 0.05)

Four x*Brassicoraphanus* lines BB#4, BB#6, BB#12 and BB#50 were previously generated from a cross between commercial cultivars of *B. rapa* and *R. sativus* followed by microspore culture and colchicine treatment (Fig. 1a; Lee et al. 2002, 2011). All four x*Brassicoraphanus* lines showed very similar phenotypes from young seedling to adult stage (Fig. 1b). However, flower color was pale yellow in BB#6, while flowers of the other three lines were white (Fig. 1b). To assess variability in seed formation rate, x*Brassicoraphanus* lines were self-pollinated in the glasshouse, and normal and aborted seeds in siliques were counted. Average numbers of total seeds per silique were 3.38 ± 0.21 in BB#4, 2.20 ± 0.26 in BB#6, 1.97 ± 0.18 in BB#12 and 2.32 ± 0.21 in BB#50 (Fig. 2a). Proportions of normal seeds per silique were 52.23 ± 2.42% in BB#4, 52.51 ± 6.17% in BB#6, 72.20 ± 1.56% in BB#12 and 81.25 ± 3.34% in BB#50 (Fig. 2b). Although production of normally developed seeds did not differ significantly among x*Brassicoraphanus* lines (ANOVA, *p* = 0.418), the aborted seed ratios were significantly lower in BB#12 and BB#50 than in BB#4 and BB#6 (ANOVA with Duncan’s multiple test, *p* = 4.53 × 10^–04^). These data suggest that each x*Brassicoraphanus* line has similar phenotypes in most traits but substantially differs for seed production efficiency.

### Differential pollen viability in x*Brassicoraphanus*

**Fig. 3:**
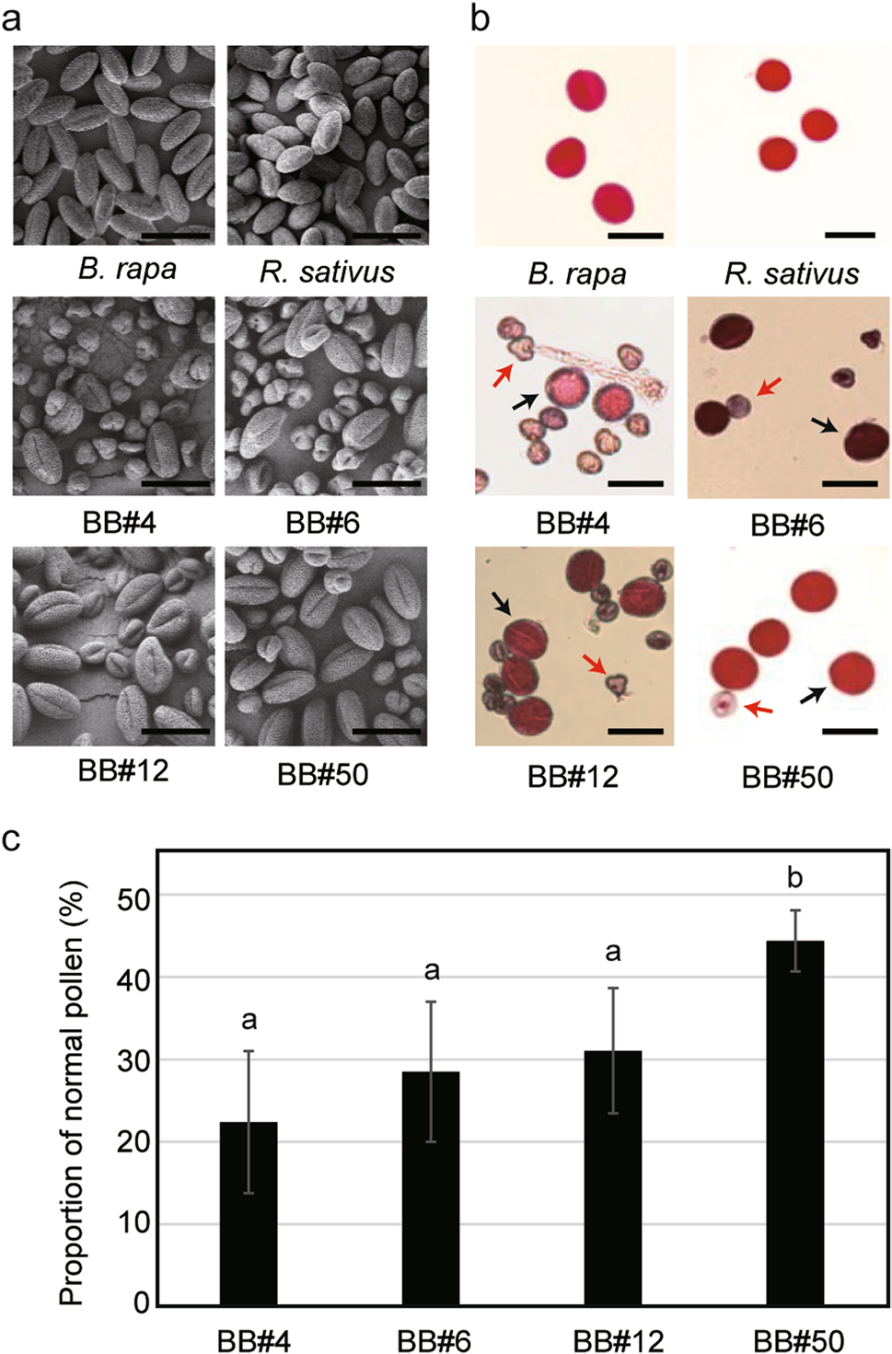
Variations of pollen viability in x*Brassicoraphanus* lines. **a** Shapes of pollen grains in *B. rapa*, *R. sativus*, BB#4, BB#6, BB#12 and BB#50. Scale bar = 50 μm. **b** Pollen grains stained with Alexander’s staining solution. Black and red arrows represent viable and non-viable pollen, respectively. Scale bar = 50 μm. **c** Proportion of normal pollen. More than one thousand pollen grains from four independent plants were analyzed for each line. Error bars indicate standard deviations, and differences among x*Brassicoraphanus* lines were tested with one-way ANOVA, as indicated with letters, by Duncan’s multiple test (*p* < 0.05)

Pollination is the first step in sexual reproduction, and thus, fertile pollen grains are required for fertilization and seed development. To investigate the fertility of male gametophytes in BB#4, BB#6, BB#12 and BB#50, pollen shapes were observed by SEM (Fig. 3a), and the viability was determined by staining pollen with Alexander’s stain solution (Fig. 3b). All normal pollen grains of *B. rapa* and *R. sativus* have a prolate spheroid or tricolpate shape, in accordance with previous studies (Goda 2018; Hossain et al. 1990), but two different types of pollen grains were also observed in all x*Brassicoraphanus* lines. One pollen type had a spheroid shape phenotypically similar to the parental species, while the other displayed a spherical shape and the grains were noticeably smaller (Fig. 3a). When stained for viability with Alexander’s solution, large spheroid pollen grains were all stained in red, whereas the deformed and shrunken pollen grains of x*Brassicoraphanus* lines remained unstained, indicative of pollen sterility. By counting the number of normal and abnormal pollen grains in the x*Brassicoraphanus* lines, we determined the proportion of normally shaped pollen to be 22.38 ± 8.61% in BB#4, 28.50 ± 8.50% in BB#6, 31.04 ± 7.59% in BB#12 and 44.39 ± 3.70% in BB#50 (Fig. 3c), revealing approximately a twofold greater viability in BB#50 than BB#4 (ANOVA with Duncan’s multiple test, *p* = 8.95 × 10^−4^). This observation suggests that superior pollen viability observed in BB#50 might be associated with a higher normal seed development rate for reproductive success.

### Formation of micronuclei in x*Brassicoraphanus* lines BB#4 and BB#6

**Fig. 4:**
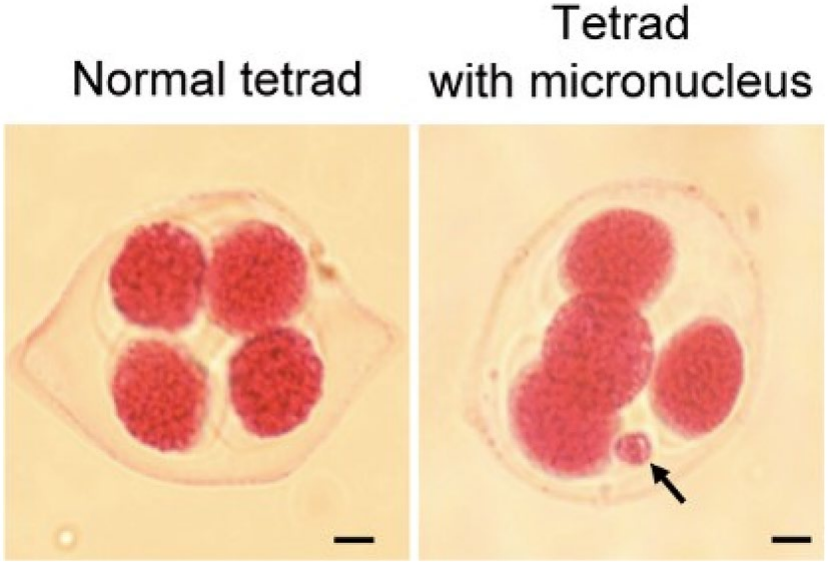
Tetrad and micronucleus formation in x*Brassicoraphanus* PMCs. A normal tetrad (left) and a tetrad with a micronucleus (right) are shown. Arrow indicates the micronucleus. Scale bar = 10 μm

At the end of microsporogenesis, microspores are released from the tetrad and develop into mature pollen. Thus, proper tetrad formation should precede microgametogenesis to produce functional pollen. To investigate the formation of tetrads in x*Brassicoraphanus* PMCs, male gametophytes from buds 1–2 mm in size were stained with Alexander’s staining solution. Most tetrads in x*Brassicoraphanus* lines consisted of four individual microspores, but additional micronuclei were frequently observed in a few cases (Fig. 4a). The frequencies of tetrads containing micronuclei were 6.98% in BB#4, 4.79% in BB#6, 2.28% in BB#12 and 0.74% in BB#50 (Table 1). It is presumed that microspores released from unbalanced tetrads are unable to develop into normal pollen grains due to an incomplete chromosome complement, and production of abnormal microspores is likely to cause a reduction in pollen fertility observed in x*Brassicoraphanus* lines BB#4 and BB#6.

**Table 1:** Frequency of tetrads with micronucleus

Lines	Normal tetrad %	Tetrad with micronucleus %
BB#4	93.02 (240/258)^a^	6.98 (18/258)^b^
BB#6	95.21 (338/355)^a^	4.79 (17/355)^b^
BB#12	97.72 (472/483)^a^	2.28 (11/483)^b^
BB#50	99.26 (404/407)^a^	0.74 (3/407)^b^

^a^Number of normal tetrads/number of observed PMCs

^b^Number of tetrads with micronucleus/number of observed PMCs

Indeed, a positive relationship was revealed between micronucleus formation and pollen abnormality in these synthetic x*Brassicoraphanus* lines (Pearson’s correlation coefficient, *r* = 0.9222, *p* = 0.0389, one-tailed test), suggesting that increased pollen viability observed in BB#50 might result from less micronuclei formation during microsporogenesis.

### Abnormal meiotic chromosome behaviors during microsporogenesis in x*Brassicoraphanus* PMCs

**Fig. 5:**
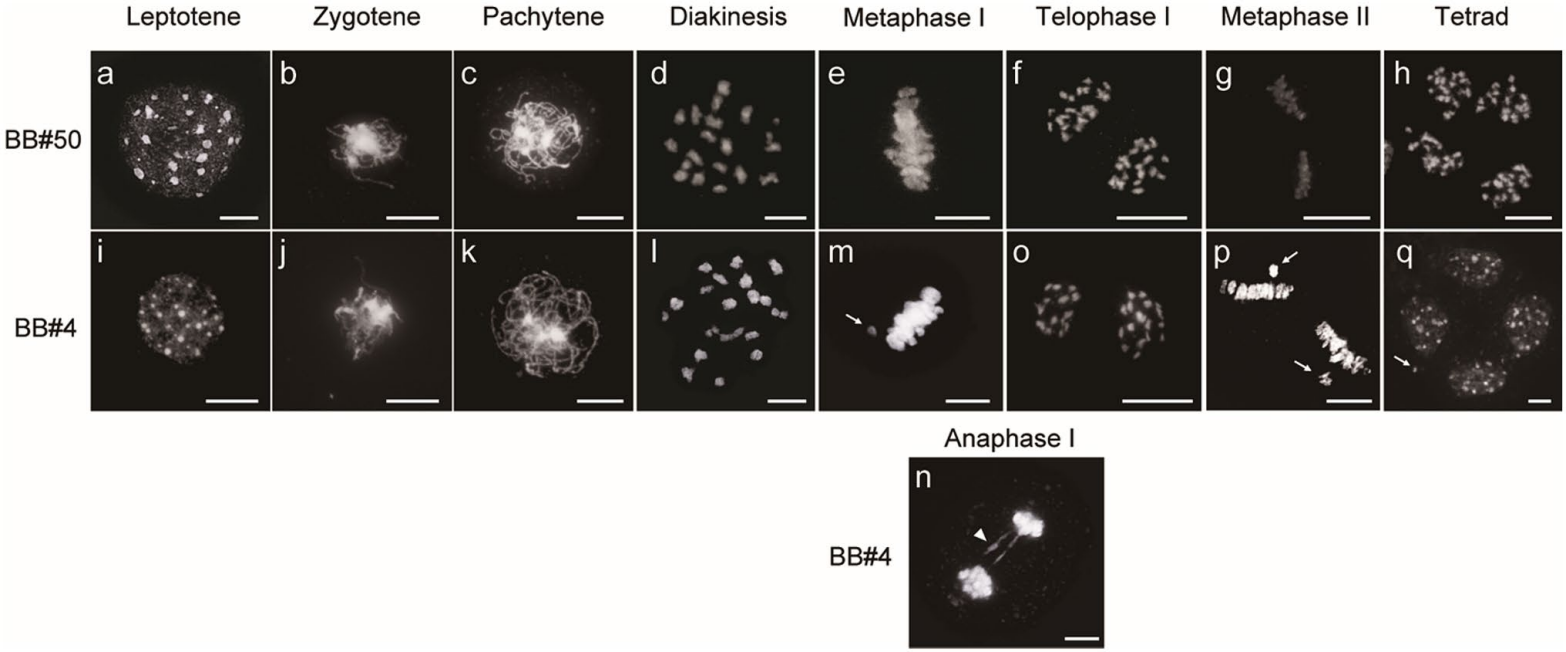
Meiotic chromosome behaviors in PMCs of BB#50 and BB#4. The chromosomes in PMCs of BB#50 (**a–h**) and BB#4 (**i**–**q**) were stained with DAPI. Chromosomes were condensed at leptotene (**a** and **i**) and thin threads were observed at zygotene (**b** and **j**). The synapsis was formed at pachytene (**c** and **k**) and bivalents produced at diakinesis (**d** and **l**). All chromosomes were located at the central plate at metaphase I (**e**) and separated at telophase I (**f** and **o**). All chromosomes were aligned at the central plate at metaphase II (**g**). Four microspores were produced at the tetrad stage (**h** and **q**). Univalent chromosomes (white arrows) and chromosome bridges (arrowhead) were observed at metaphase I, anaphase I, metaphase II and tetrad stages of BB#4 (**m**, **n**, **p** and **q**). Scale bar = 10 μm

In many newly synthesized interspecific hybrids, abnormal meiotic chromosome behaviors were associated with non-homologous chromosome interactions, resulting in unbalanced gamete formation and reduced fertility (Martinez-Perez and Colaiacovo 2009). To investigate the cause of micronucleus formation observed in x*Brassicoraphanus* lines, we compared meiotic chromosomal behaviors in PMCs of BB#50 and BB#4 (Fig. 5). At leptotene, the first substage of prophase I, chromosome condensation was initiated (Fig. 5a, i), and pairing of homologous chromosomes occurred at zygotene (Fig. 5b, j). At pachytene, homologous chromosomes were synapsed (Fig. 5c, k), and a total of 19 bivalents produced at diakinesis (Fig. 5d, l). Bivalents were aligned at the central plate at metaphase I (Fig. 5e) and separated at telophase I (Fig. 5f, o). At metaphase II, sister chromatids were placed at the equatorial plate (Fig. 5g), and the second meiotic division was completed. Finally, four daughter cells were produced at the tetrad stage (Fig. 5h).

Throughout the entire course of meiosis, normal chromosome behaviors were observed in most PMCs of BB#50. However, it is notable that univalent chromosomes were sporadically detected at both metaphase I and II (Fig. 5m, p), and notably, an interchromosomal bridge was occasionally formed at anaphase I in BB#4 PMCs (Fig. 5n). Therefore, dysregulation of meiosis such as the formation of univalents and inter-chromosomal bridges may cause chromosome aberrations (Fig. 5q), and these abnormalities in chromosome segregation may induce the formation of unbalanced tetrads or polyads. In BB#50, a normal meiotic chromosome behavior was observed in the majority of PMCs, whereas 25.0% (*n* = 48), 25.0% (*n* = 24) and 33.3% (*n* = 6) of PMCs in BB#4 displayed abnormal chromosome behaviors at metaphase I, anaphase I and metaphase II, respectively (Table 2). This analysis demonstrates that abnormal segregation of meiotic chromosomes occurred more frequently in BB#4 compared to BB#50 (Fisher’s exact test, *p* = 6.85 × 10^−6^) (Table 2). These results also suggest that micronucleus formation observed in BB#4 is tightly associated with preceding meiotic segregation abnormality, affecting subsequent microspore development.

**Table 2:** Frequency of abnormal meiosis in BB#4 and BB#50

Stage	BB#4		BB#50	
	Abnormality %	Normality %	Abnormality %	Normality %
Metaphase I	25.0 (12/48)^a^	75.0 (36/48)^b^	1.9 (1/52)^a^	98.1 (51/52)^b^
Anaphase I	25.0 (6/24)^a^	75.0 (18/24)^b^	0 (0/18)^a^	100 (18/18)^b^
Metaphase II	33.3 (2/6)^a^	66.7 (4/6)^b^	0 (0/4)^a^	100 (4/4)^b^

^a^Number of PMCs showing abnormal meiosis/number of observed PMCs

^b^Number of PMCs showing normal meiosis/number of observed PMCs

### Identification of the parental origin of univalent chromosomes

**Fig. 6:**
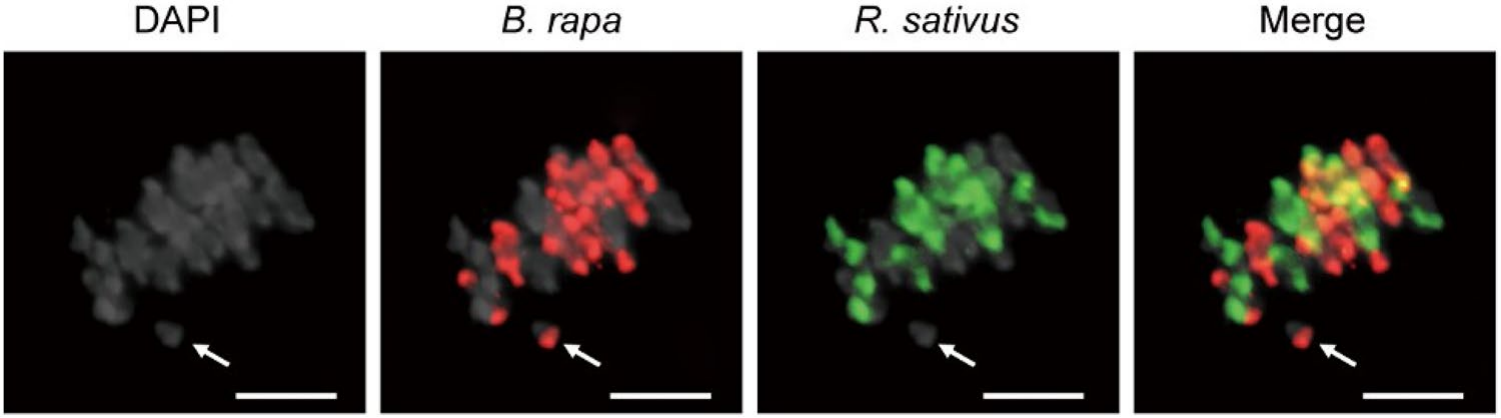
Univalent formation at metaphase I in the BB#4 PMC. Genomic DNA isolated from *B. rapa* and *R. sativus* was labeled with digoxigenein-11-dUTP (red) and biotin-16-dUTP (green) by nick translation, respectively, and used as a probe for GISH analysis. An arrow indicates a univalent chromosome. Scale bar = 10 μm

The univalent chromosomes induced by a failure of precise homologous chromosome interactions as observed in BB#4 (Fig. 5) may produce micronuclei. The parental origins of chromosomes of BB#4 were investigated by GISH analysis. PMCs displaying abnormal chromosome behaviors at metaphase I were collected, and chromosomes derived from *B. rapa* and *R. sativus* were stained in red and green, respectively (Fig. 6). The GISH analysis identified that 93.75% (15/16 PMCs) of univalent chromosomes in BB#4 had origins of *B. rapa*. This observation suggests that, as in many newly synthesized allopolyploids, a subset of parent-of-origin-specific chromosomes may preferentially participate in non-homologous pairing and cause abnormal segregation during gamete production.

## Discussion

Interspecific hybridization has been utilized in plant breeding to transfer desirable traits between species, producing novel phenotypes with the desire of heterosis. Although several spontaneous interspecific hybrids have occurred in nature as exemplified by the model of ‘triangle of U’, artificially synthesized hybrid plants frequently exhibit reduced viability and sterility (Bing et al. 1996; García-Fortea et al. 2019; Monteiro et al. 2011; Warwick et al. 2003). The failures of hybrids to produce viable seeds or fertile pollen grains are due in part to the hybridization barrier which is a major hindrance to hybrid breeding. Hybridization barriers are categorized into pre- and post-zygotic barriers according to the timing of fertilization. Pre-zygotic barriers are engaged prior to fertilization between egg and sperm within the embryo sac, mostly preventing pollen germination and/or pollen tube growth and guidance. Post-zygotic barriers act after fertilization and are usually manifested as poor development of the zygote and sterility of the hybrid offspring.

In this study, we observed several reproduction-related defects which may signify post- zygotic barriers in synthetic x*Brassicoraphanus* lines. Few morphological differences were observed among them, but seed development and pollen viability were significantly different between lines BB#50 and BB#4 (Figs. 2, 3), suggesting that post-zygotic barriers were unequally expressed in each offspring. Seed abortion in hybrids is likely caused by a failure in endosperm development or by dysregulation of embryogenesis in the hybrid zygote (Kradolfer et al. 2013; Lafon-Placette et al. 2017; Rebernig et al. 2015).

Importantly, hybrid sterility is often associated with abnormal meiosis during gamete formation (Meena et al. 2017; Mendes-Bonato et al. 2007; Ohmido et al. 2015). It was previously reported that newly synthesized *B. napus* are frequently burdened with massive chromosome rearrangements such as translocation, deletion, insertion and aneuploidy (Gaeta et al. 2007; Xiong et al. 2011). Abnormalities in meiosis associated with poor pollen viability are also reported in a variety of synthetic hybrid plants (Meena et al. 2017; Mendes-Bonato et al. 2007; Ohmido et al. 2015). In our cytological analysis, abnormal chromosome segregation was intermittently observed in PMCs of BB#4 (Fig. 5; Table 2), with a more frequent formation of micronuclei than in BB#50 (Fig. 4; Table 1). The micronucleus is a small nuclear mass containing DNA and surrounded by a membrane, which can be generated from an isolated chromosome or chromosome fragments. Therefore, tetrads containing micronuclei may represent unbalanced chromosome complements, consequently imposing detrimental effects on subsequent pollen development. In our study, the formation of micronuclei in x*Brassicoraphanus* lines was positively correlated with pollen deformation, suggesting that the persistence of micronuclei induced by a meiotic failure severely compromised pollen viability observed in BB#4.

The chromosomes forming univalents at metaphase I and II in x*Brassicoraphanus* PMCs were mostly derived from *B. rapa* (93.75%) (Fig. 6). This observation suggests that specific chromosomes or fragments might trigger non-homologous interactions, resulting in meiotic abnormality during chromosome segregation. In resynthesized *B. napus*, aneuploidy was frequently caused by homoeologous interactions between the chromosomes sharing a high level homology in sequence and structure. For instance, syntenies of A1 and C1, and A2 and C2 chromosomes (A from *B. rapa* and C from *B. oleracea*) are highly conserved (Parkin et al. 2005), and a deletion or insertion of such chromosomes was highly recurrent in early generations of resynthesized *B. napus* (Xiong et al. 2011). In the genome of x*Brassicoraphanus*, albeit an intergeneric hybrid between distantly related species, chromosomes A8 and R8 have syntenic homology (Park et al. 2020). This suggests the possibility that univalent chromosomes in BB#4 are generated by the persistence of non-homologous interactions, even between parental chromosomes. In the end, stabilization of hybrid genome of x*Brassicoraphanus* may require an additional process at the chromatin level, for which epigenetic modifications such as DNA methylation and chromatin remodeling should play an important role to suppress undesirable stand exchanges between non-homologous chromosomes during meiosis.
